# Comprehensive analysis of T-cell regulatory factors and tumor immune microenvironment in stomach adenocarcinoma

**DOI:** 10.1186/s12885-024-12302-w

**Published:** 2024-05-07

**Authors:** Shuchang Wang, Weifeng Zhang, Xinrui Wu, Zhu Zhu, Yuanbiao Chen, Wangrui Liu, Junnfei Xu, Li Chen, Chun Zhuang

**Affiliations:** 1 Department of Gastrointestinal Surgery, Renji Hospital, Shanghai Jiao Tong University School of Medicine, Shanghai, 200127 China; 2 Department of Cardiology, Shanghai Chest Hospital, Shanghai Jiao Tong University School of Medicine, Shanghai, 200127 China; 3 Department of Urology, Renji Hospital, Shanghai Jiao Tong University School of Medicine, Shanghai, 200127 China; 4 Department of Clinical Medicine, Medical School of Nantong University, Nantong, China; 5 Department of Neurosurgery, Affiliated Hospital of Youjiang Medical University for Nationalities, Baise, China; 6 Department of Interventional Oncology, Renji Hospital, Shanghai Jiao Tong University School of Medicine, Shanghai, 200127 China; 7 Department of General Surgery, Affiliated Hospital of Nantong University, Nantong, 226001 Jiangsu China; 8 Department of Nursing, Renji Hospital, Shanghai Jiao Tong University School of Medicine, Shanghai, 200127 China

**Keywords:** Prognostic marker, Gastric Cancer, Gene Signature, Immune Infiltration, Immunotherapy

## Abstract

**Background:**

Gastric cancer (GC) is one of the most prevalent malignant tumors worldwide and is associated with high morbidity and mortality rates. However, the specific biomarkers used to predict the postoperative prognosis of patients with gastric cancer remain unknown. Recent research has shown that the tumor microenvironment (TME) has an increasingly positive effect on anti-tumor activity. This study aims to build signatures to study the effect of certain genes on gastric cancer.

**Methods:**

Expression profiles of 37 T cell-related genes and their TME characteristics were comprehensively analyzed. A risk signature was constructed and validated based on the screened T cell-related genes, and the roles of hub genes in GC were experimentally validated.

**Results:**

A novel T cell-related gene signature was constructed based on CD5, ABCA8, SERPINE2, ESM1, SERPINA5, and NMU. The high-risk group indicated lower overall survival (OS), poorer immune efficacy, and higher drug resistance, with SERPINE2 promoting GC cell proliferation, according to experiments. SERPINE2 and CXCL12 were significantly correlated, indicating poor OS via the Youjiang cohort.

**Conclusions:**

This study identified T cell-related genes in patients with stomach adenocarcinoma (STAD) for prognosis estimation and proposed potential immunotherapeutic targets for STAD.

**Supplementary Information:**

The online version contains supplementary material available at 10.1186/s12885-024-12302-w.

## Background

As one of the most prevalent malignant tumors worldwide, gastric cancer (GC) has high morbidity and mortality rates, with stomach adenocarcinoma (STAD) as one of the major pathological subtypes [1]. STAD accounts for 80–90% of all gastric cases [2]. The 5-year survival rate of patients with STAD with advanced or metastatic disease is less than 30% [3, 4]. Most patients worldwide are not diagnosed until the advanced stage owing to the absence of significant early symptoms and limitations in medical screening [5, 6]. Therefore, the accurate prediction of biomarkers is crucial for clinical prognostication and treatment. Currently, surgical resection is the primary treatment for STAD [7]. However, because of the recurrence and metastasis of STAD after surgery, its prognosis is poor [8]. Immunoassay inhibitors of PD-L1 have positive effects on immunotherapy and targeted therapy, and they can significantly improve the prognosis of patients with stable microsatellite carcinomas [9].

Increasing evidence suggests that the tumor microenvironment (TME) plays a role in anti-tumor activity and contributes to the prediction of immune checkpoint blockade (ICB) responses [10–13]. However, gaining advantages from STAD is not common [14]. The TME comprises tumor cells, fibroblasts, extracellular matrix elements, and diffusing cytokines [15, 16]. Growing tumor cells can be recognized and destroyed by tumor-infiltrating immune cells. This defensive behavior may involve inflammation as tumor cells evade immune defense mechanisms by selecting more aggressive tumor clones [17]. High levels of regulatory T cells are commonly found in patients with cancer and are associated with the prognosis of different types of STAD [18].

Furthermore, treatment is influenced by T-cell therapy [19]. However, these cellular treatments need to be improved further to improve the cure rate. To date, studies on T-cell function have mainly focused on the negative regulatory factors contributing to functional deficiency [20]. The US Food and Drug Administration (FDA) approved the first chimeric antigen receptor T-cell (CAR-T) therapy [21]. Nevertheless, the therapeutic effect of the chimeric antigen receptor on STAD did not meet expectations because of the failure of T cells to perform their effector function within the TME fully. Positive regulators reportedly have a positive effect on T-cell proliferation, activation, and secretion of key cytokines, thereby optimizing and improving T-cell function in STAD [22], that may further improve the treatment of STAD.

In this work, we aim to build signatures to study the effect of certain genes on gastric cancer using LASSO and multivariate Cox regression analyses. Our findings underscore the significance of considering tumor-infiltrating immune cells and their interactions with the TME in prognostic assessment and treatment planning. Also, the identification of potential therapeutic targets such as CXCL12 and SERPINE2 presents opportunities for developing targeted interventions aimed at mitigating immune exhaustion and enhancing treatment efficacy.

## Materials and methods

### STAD data source and preprocessing

Data on STAD-related gene expression, prognosis, and clinicopathological characteristics were collected from comprehensive GEO (GSE38749, GSE84437, GSE34942, and GSE15459; https://www.ncbi.nlm.nih.gov/geo/) and TCGA (https://portal.gdc.cancer.gov/) datasets. Following this, the FPKM values were converted to TPM values within the composite matrix. The 37 T cell-related genes were derived from the latest research [22]. Ten tumors and ten normal single-cell ribonucleic acid (scRNA) samples were obtained from GSE18394.

### WGCNA co-expression network construction

The co-representation network of differentially expressed genes (DEG) is built by the “WGCNA”package in the R package (version xx; The R Foundation for Statistical Computing, Vienna, Austria). Subsequently, pairs of genes were subjected to Pearson’s correlation matrix analysis.

To further analyze sample clustering for outliers detection, we calculated the similarity of the characteristic genes within each module, selected the standard tangent value from the module tree, and merged specific modules. The module-feature relationship between modular feature genes and T cells was described within the characteristic gene network, and two modules related to specific traits were found.

### DEGs identification and enrichment analyses

The parameter of “limma”in the R package (The R Foundation for Statistical Computing, Vienna, Austria) was set to 1.5, and the P-value was adjusted to be less than 0.05 to screen DEGs across different T-cell clusters. A total of 178 genes were identified as DEGs. Moreover, disease ontology (DO), gene ontology (GO), and Kyoto Encyclopedia of Genes and Genomes (KEGG) enrichment analyses were used to explore the characteristics of the DEGs [23–25].

### Construction of prognostic T cell-related signature

The signature score was calculated to express the properties of each cell type, and DEGs were identified from T-cell clusters using LASSO and multivariate Cox regression analyses. Univariate Cox regression, overall survival (OS) (*p* < 0.05), and heatmap analysis were used to verify the signature validity. The GSE38749, GSE84437, GSE34942, GSE15459, and TCGA-STAD cohorts were segmented into training (*n* = 522) and test (*n* = 521) datasets at a ratio of 1:1, and the former was used to establish risk characteristics. We analyzed the various trajectories of each variable and identified candidate genes using multivariate Cox analysis. The risk score was calculated using the following formula:

Risk score = Σ(Expi × Coefi).

### Analysis of TME, immunological checkpoint, mutation, and drug susceptibility

Based on the data from TCIA and TIDE (http://tide.dfci.harvard.edu/), a boxplot was used to visualize the differences between the two groups in the expression levels and treatment diversity of the PD-1 and CTLA-4 immunological checkpoints. Additionally, we examined drug susceptibility and mutation status in the high- and low-risk groups using the prophetic and maftools packages, respectively.

### Validation of external cohort

Survival analysis was conducted on the external IMV210 cohort and GSE62254 for validation. This involved analyzing the binary response in risk scores among CR/PR and SD/PD, as well as Kaplan–Meier survival analysis between high-risk and low-risk score groups. For the scRNA data, we created a project using the Seurat package and set the following screening criteria: (1) Each gene was expressed in at least three cells; (2) The total number of molecules detected in each cell was > 1000; (3) The number of genes detected in each cell was > 200 and < 10,000; (4) The proportion of mitochondrial and ribosomal genes was < 20%. The first 2000 highly variable genes were selected for scale analysis, and PCA dimensionality reduction analysis was performed. Twenty PC were selected for unsupervised clustering and labeled according to the marker genes of different cell types. Four methods, Ucell, singscore, ssgsea, and addmoduleScore, were used to score the TME of both normal and tumor tissues. The scores obtained from the UCell, irGSEA, and GSVA packages were utilized to explore the differential expression of SEPRINE2 in T cells from normal and tumor groups.

### Youjiang cohort and Immunohistochemical

OS with high and low SEPRINE2 expression in GC was verified using the Youjiang cohort, and immunohistochemistry was conducted to test the content of high or low-expression SEPRINE2 samples in formalin/PFA-fixed paraffin-embedded sections (*n* = 93). Human GC tissues were stained for SERPINE2/PN-1 using ab154591 at a dilution of 1/500.

### Cell culture and transfection

Human GC cell lines AGS and BGC-823 were purchased from the Shanghai Institutes of Biological Sciences (Shanghai, China). AGS and BGC-823 cells were cultured in RPMI 1640 medium (Gibco, USA) supplemented with 1% penicillin-streptomycin (Gibco, USA) and 10% fetal bovine serum (Gibco, USA). BGC-823 cells were maintained in Dulbecco’s modified DMEM medium (Gibco, USA) supplemented with 1% penicillin-streptomycin (Gibco, USA) and 10% fetal bovine serum (Gibco, USA). All the cells were cultured at 37 °C and 5% CO2. Cell transfections were performed using Lipofectamine 3000 (Invitrogen, USA), with oligonucleotides as a control. After 48 h of transfection, cellular RNA and proteins were extracted.

### Cell migration assays and proliferation

Stably transfected AGS and BGC-823 cells were seeded in 96-well plates at a density of 5 × 104 cells/mL. A Cell Counting Kit-8 (Dojindo, Japan) was used to test cellular proliferative capacity. On each of the subsequent 6 days, the optical density was evaluated at 450 nm using a microplate reader (TEAN, Switzerland). Additionally, a transwell migratory assay was conducted to study the migratory response of AGS and BGC-823. The cell density was standardized to 2 × 105 cells/mL, and a volume of 100 µL cell suspension was added to the upper chamber. Medium containing 20% fetal bovine serum was added to the lower chamber. After 24 h, AGS and BGC-823 cells within the lower chamber were washed in 4% polyoxymethylene for 15 min, followed by staining with 0.1% crystal violet and subsequent rinsing with deionized water for 30 min. Finally, the cells were counted under a microscope.

### Statistical analyses

R (version 4.1.2, R Foundation for Statistical Computing, Vienna, Austria) (https://www.r-project.org/) was applied for all analyses, and statistical significance was set at *p* < 0.05.

## Results

### Landscape of T cell-related gene modification in STAD

**Fig. 1:**
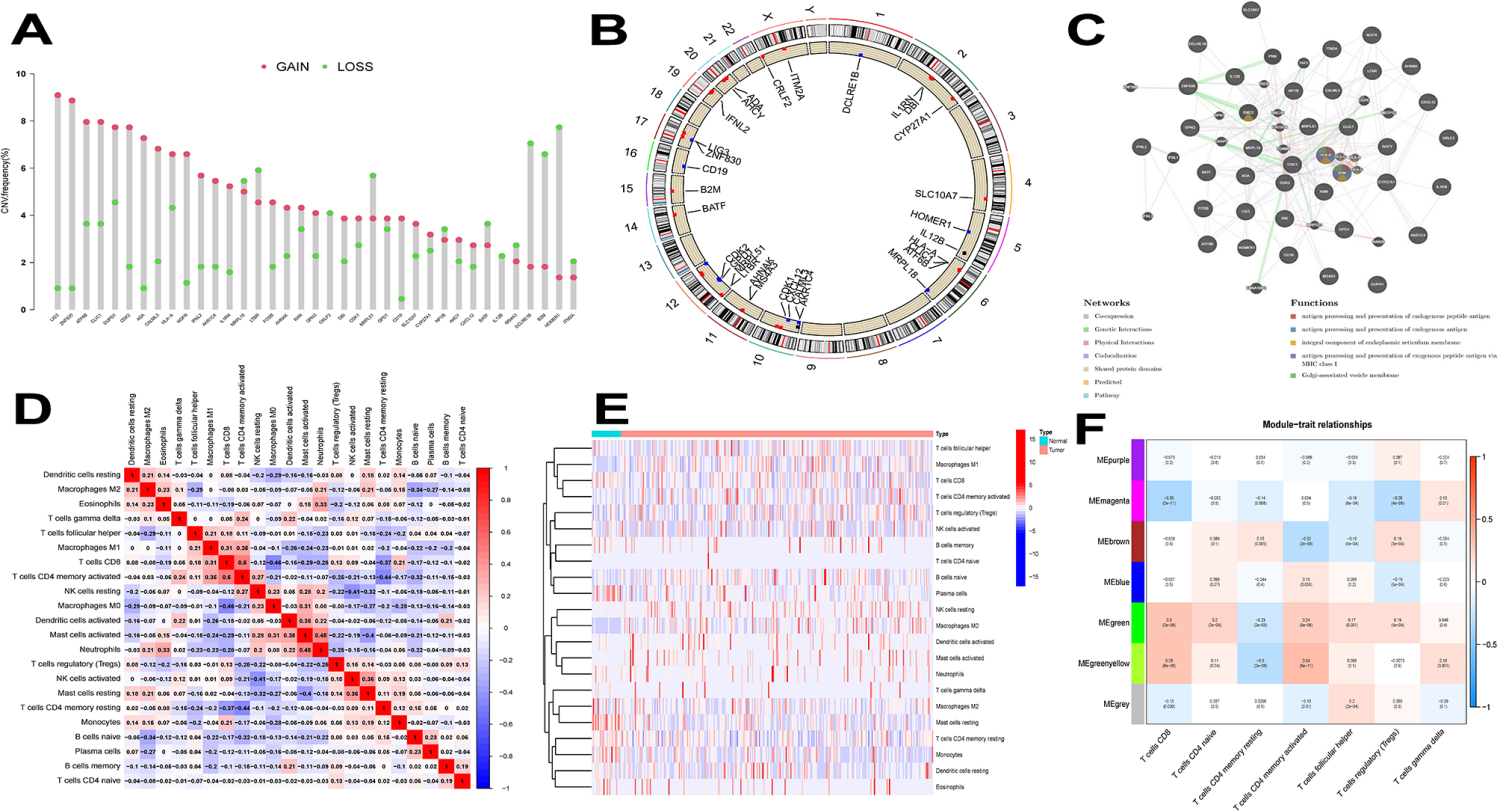
Genetic alterations and tumor microenvironment (TME) of T-cell related genes in stomach adenocarcinoma (STAD). **a** Frequencies of copy number variations (CNV) gain, loss, and non-CNV among T-cell related genes. **b.** Locations of CNV alterations in T-cell related genes on 23 pairs of chromosomes. **c.** The interaction between 37 T-cell related genes in STAD. **d.** Correlation matrix for all 22 immune cell proportions. The darker the colour, the higher the correlation was. **e.** Heatmap of the 22 immune cell proportions. **f.** Module − trait relationships between module eigengenes and T-cells

Among the included 37 T cell-related genes, the copy number variations (CNV) were investigated (Fig. 1a). Notably, significant increases in CNV were observed in genes such as LIG3, ZNF830, ATF6B, CLIC1, and DUPD1, while decreases were most obvious in HOMER1, DCLRE1B, B2M, MRPL51, and LTBR. The distribution profile of CNV modification in the 37 T cell-related genes is illustrated in Fig. 1b. To investigate the association between genes and their interactions in STAD studies, we generated a T cell-related co-expression visualization protein-protein interaction (PPI) network (Fig. 1c).

### Distribution of immune infiltration in STAD

The proportions of each T-cell subset were not significantly correlated, as shown in Fig. 1d. The population quantities with remarkably positive relevance were CD8 + T cells and CD4 + memory-activated T cells (0.5), activated neutrophils and mast cells (0.45), and resting mast cells and activated natural killer cells (0.36). As shown in Fig. 1e, the levels of macrophage M1, T-cell follicular helper, macrophage M0, and T-cell CD4 memory activation were comparatively high in the tumor samples contained in the heatmap. In the PPI network, key gene pathways such as B2M and HLA have multiple functions and play a central role in co-expression and physical interactions.

### WGCNA co-expression analysis of STAD samples

The intercept value was set to 76 to detect outliers, the outlier samples were removed, and the remaining samples were included in the analysis (Additional figure S1a). As shown in Additional figure S1b, when power = 5, the scale independence was 0.9, and the mean connectivity was relatively high. Power = 5 was set to build the co-expression module and obtain the result of the preliminary module division. Different modules were represented in different colors in line with the results of WGCNA (Additional figure S1c). To detect outliers, a tree was built using the eigenvalues of the module and very close distance module merging, with the intercept value set to 0.5 (Additonal figure S1d). As shown in Additional figure S1e, a co-expression module was constructed, and the results were obtained after merging similar modules. According to the characteristic value of each sample in each module, correlation analysis was carried out to find out two modules related to specific traits (infiltrated immune cells). Among the seven modules, the green module was highly correlated to T cells CD8 (CD8 + T cells) (R2 = 0.3; *p* = 2e-08) and activated T cells CD4 memory (R2 = 0.24; *p* = 5e-06). Furthermore, the green-yellow module showed a higher correlation with activated T cells CD4 memory (R2 = 0.34; *p* = 8e-11) and T cells CD8 (CD8 + T cells) (R2 = 0.29; *p* = 6e-08; Fig. 1f).

### Screening genes and survival analysis

**Fig. 2:**
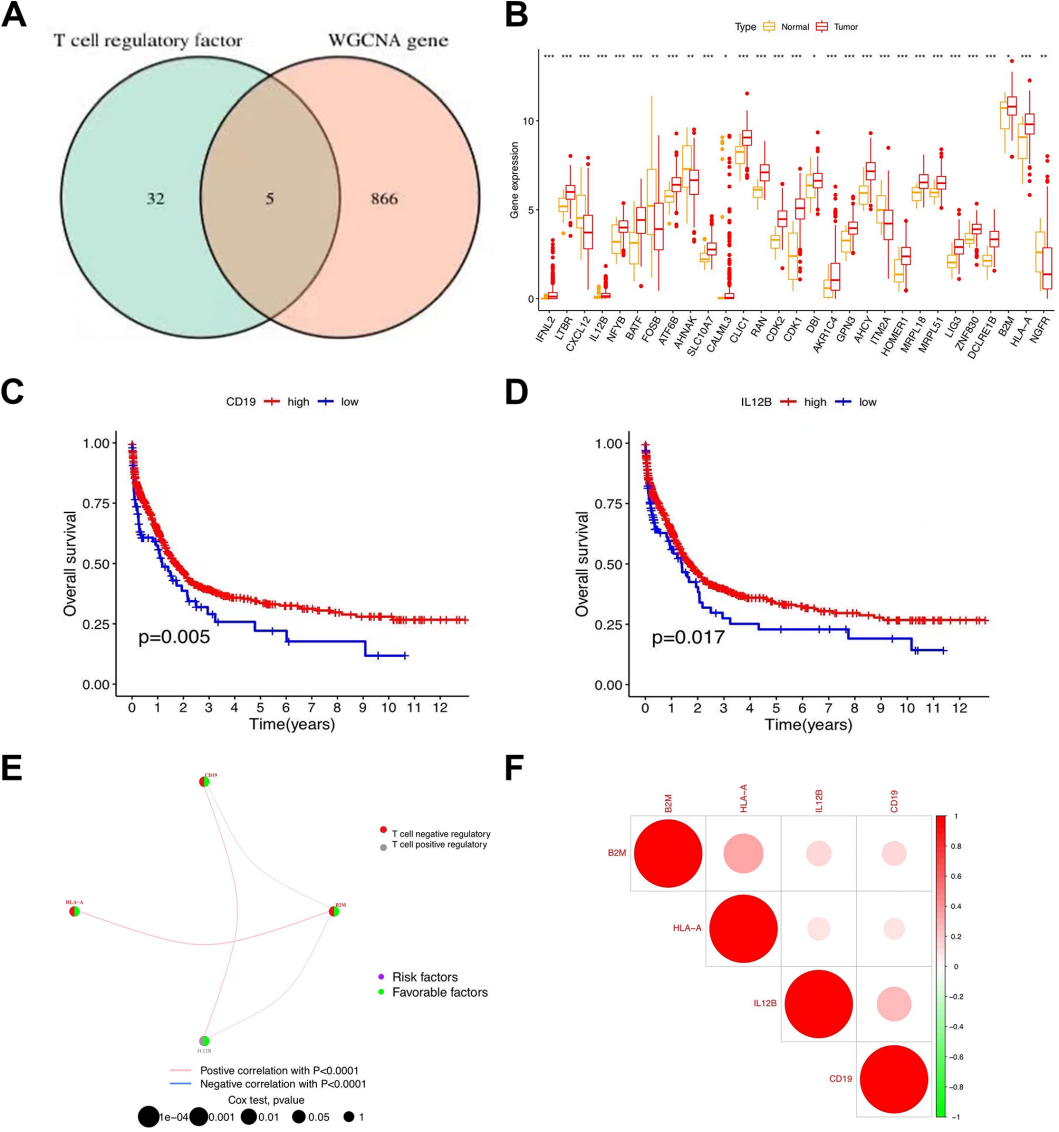
Screening genes and survival analysis. **(a)** VEEN diagram results. **(b)** Box plot demonstrating the immune cell-infiltrating characteristics of T-cell regulatory factor. **(c)** Survival analysis of genes. **(d)** The interaction between 4 genes in T-cell regulatory factor. **(e)** Correlation of genes

From the chart, we identified five genes (IFNL2, IL12B, B2M, HLA-A, and CD19) that were selected from the intersection of the T-cell regulatory factor and the WGCNA gene (Fig. 2a), with only four (IL12B, B2M, HLA-A, and CD19) expressed in selected samples. Among the T-cell regulatory factors, normal and tumor genes showed prominently distinct Inter-cell Interference Coordination (ICIC), with tumor gene expression higher than normal in RAN, CHK1, and CDK2 cells (Fig. 2b). Prognostic analysis indicated a significant survival advantage for CD19 and IL12B (Fig. 2c, d). The distribution of genes encoding T-cell regulatory factors is shown in Fig. 2e. As shown in Fig. 2f, B2M and HLA − A exhibited a high correlation, as did IL12B and CD19.

### NMF clustering based on related genes in STAD samples

**Fig. 3:**
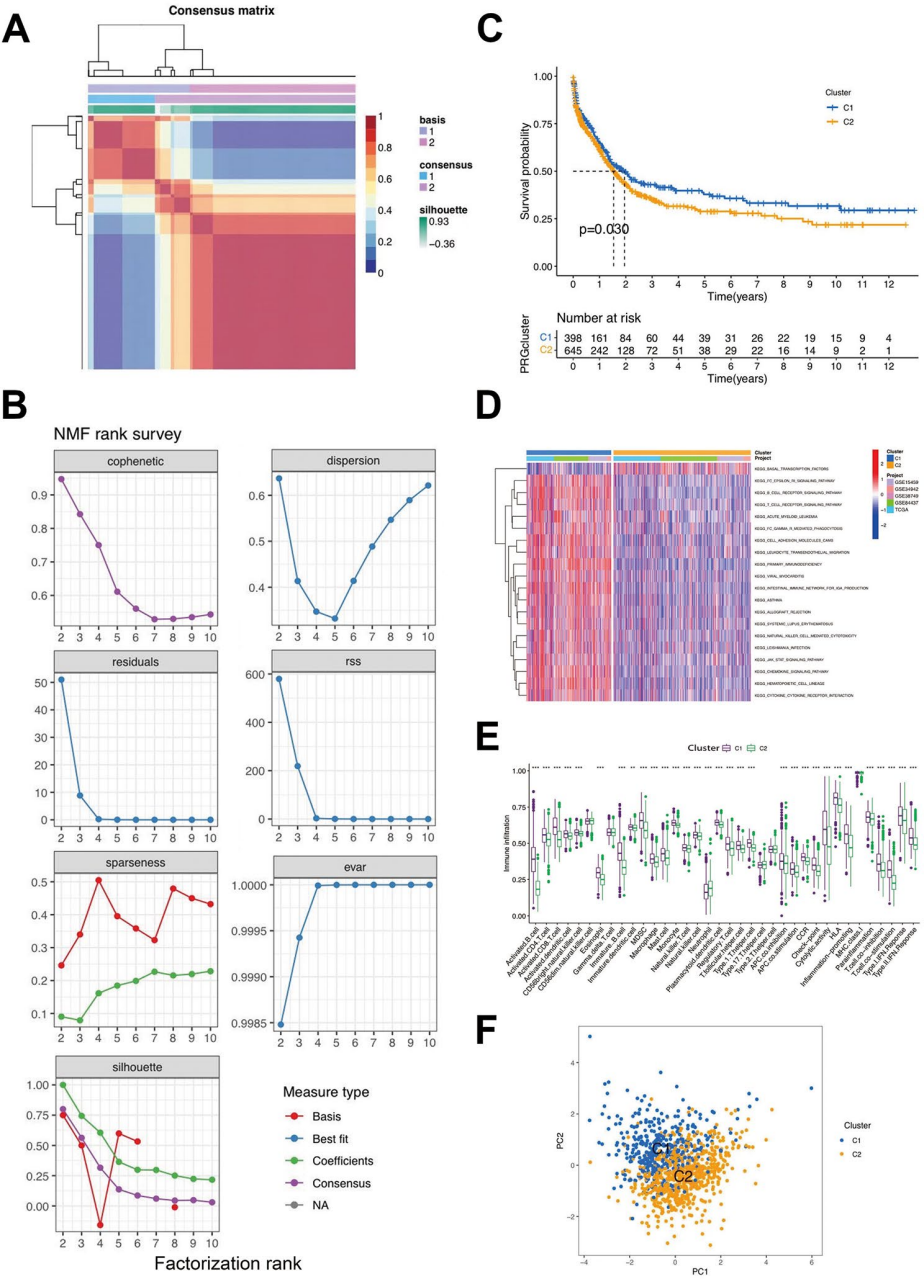
Clinicopathological and biological characteristics of two distinct T-cell clusters of samples from the combination of GSE38749, GSE84437 and The Cancer Genome Atlas-analysis of stomach adenocarcinoma (TCGA-STAD) cohorts, divided by Non-negative Matrix Factorization (NMF) analysis. **(a)** NMF analysis heatmap defining two clusters (k = 2) and their correlation area. **(b)** NMF rank survey performed on the two T-cell clusters. **(c)** Survival analysis of the two distinct clusters. **(d)** Heatmap of gene set variation analysis(GSVA) enrichment analysis. **(e)** Box plot demonstrating the immune cell-infiltrating characteristics of the two clusters. **(f)** principal components analysis(PCA) of two clusters

In the union of the GSE38749, GSE84437, GSE34942, GSE15459, and TCGA-STAD cohorts, the STAD samples were grouped into diverse molecular subtypes using NMF analysis (Fig. 3a and b). We designated these two clusters as T cell-related genes: Clusters C1 and C2. Cluster C1 displayed marked indigenous survival advantages (Fig. 3c). Furthermore, these two clusters also displayed notably diverse enrichment characteristics of the KEGG pathway in GSVA enrichment analysis. For instance, in KEGG intestinal immune network for IgA production, the KEGG T-cell receptor signaling pathway and KEGG B cell receptor signaling pathway had high-level gene expression in C1 and low-level gene expression in C2, while KEGG basal transcription factors had high expression levels in C2 and low expression levels in C1 (Fig. 3d). These two T-cell clusters also displayed marked distinctions in ICIC (Fig. 3e). Activated CD8 T-cells, activated B cells, and macrophages were amplified in cluster C1, suggesting that they could promote inflammation, while activated CD4 T-cells, type 17 T-helper cells, and neutrophils were abundant in another cluster. The results indicated that the expression of major histocompatibility complex type 1 (MHC-1) in the C1 cluster was higher than that in the other clusters, which may be one of the reasons why the survival rate of CI was higher than that of the other clusters. As shown in Fig. 3f, the genes were divided into two clusters, C1 and C2, proving that the two groups clustered well.

### GO, KEGG, and DO enrichment analyses of clusters

**Fig. 4:**
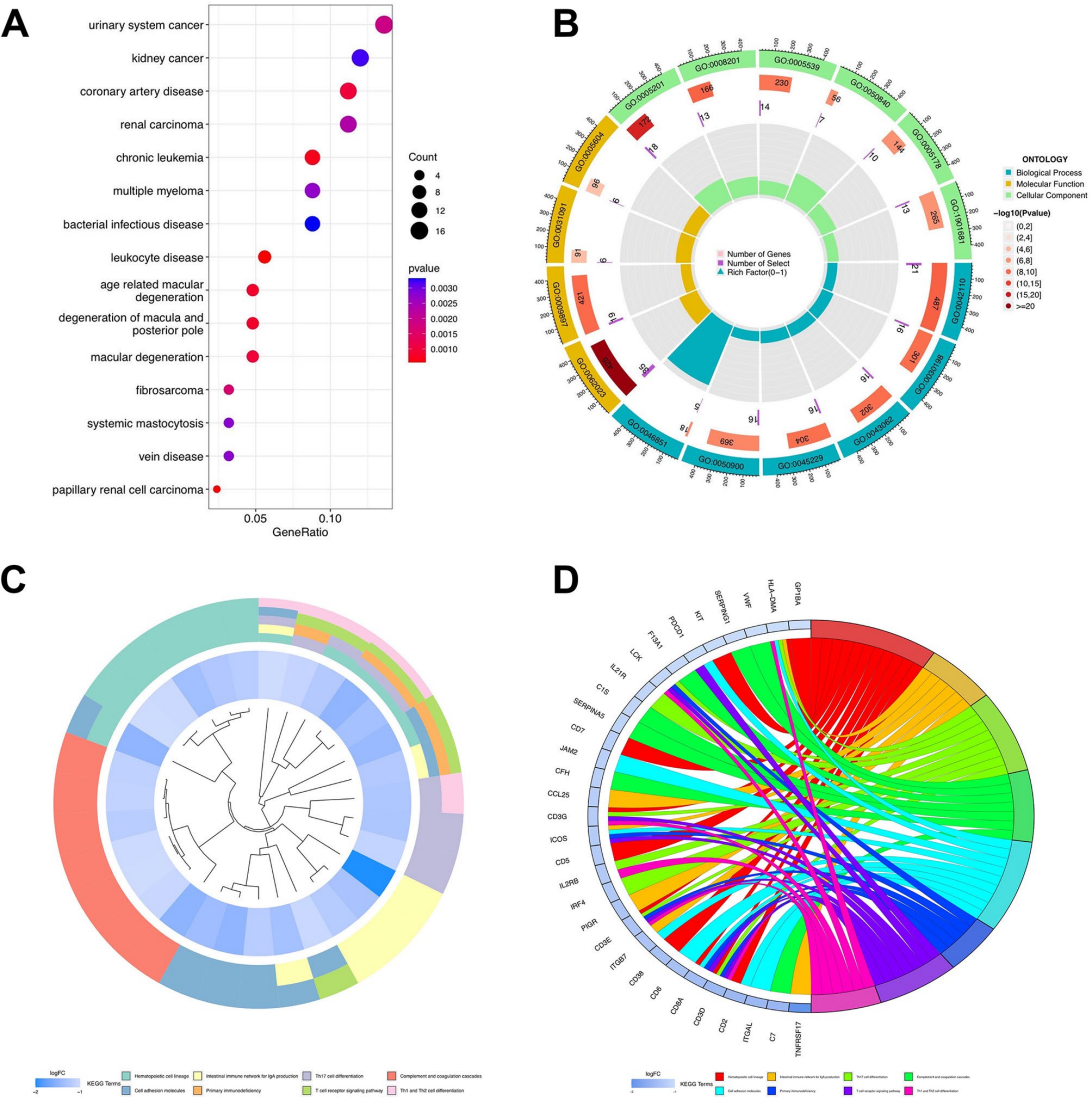
Disease Ontology (DO), Gene Ontology (GO) and Kyoto encyclopedia of genes and genomes(KEGG) enrichment analysis of T-cell related genes. **(a)** Bubble chart of DO enrichment analysis of 178 DEGs. **(b)** Enrichment circle graph of GO terms of 178 DEGs. **c-d.** Enrichment circle graph of KEGG biological process of 178 DEGs

A total of 178 genes with an absolute value of LogFC greater than 1.5 and *p* < 0.05 were obtained by analyzing the differences between the C1 and C2 groups. To investigate the association between T cell-related genes and other illnesses, DO analysis was carried out, and the results showed that all genes were strongly correlated with bacterial infectious diseases, leukocyte diseases, and systemic mastocytosis (Fig. 4a). For each T cell-related gene cluster, a deeper understanding of the characteristics and GO functional enrichment analysis was performed using the cluster package. These genes showed abnormal enrichment related to biological processes, molecular functions, and cellular components, which could also partially account for the high incidence and recurrence rates of malignant gastric cancer (Fig. 4b). Accumulation of the biological processes was studied by carrying out KEGG analysis, confirming T-cellular receptor signaling transduction pathway, Th1, Th2, and Th17 cell transdifferentiation (Fig. 4c, d), all of which are related to T-cells and the immune microenvironment.

### Establishment of the risk signature

**Fig. 5:**
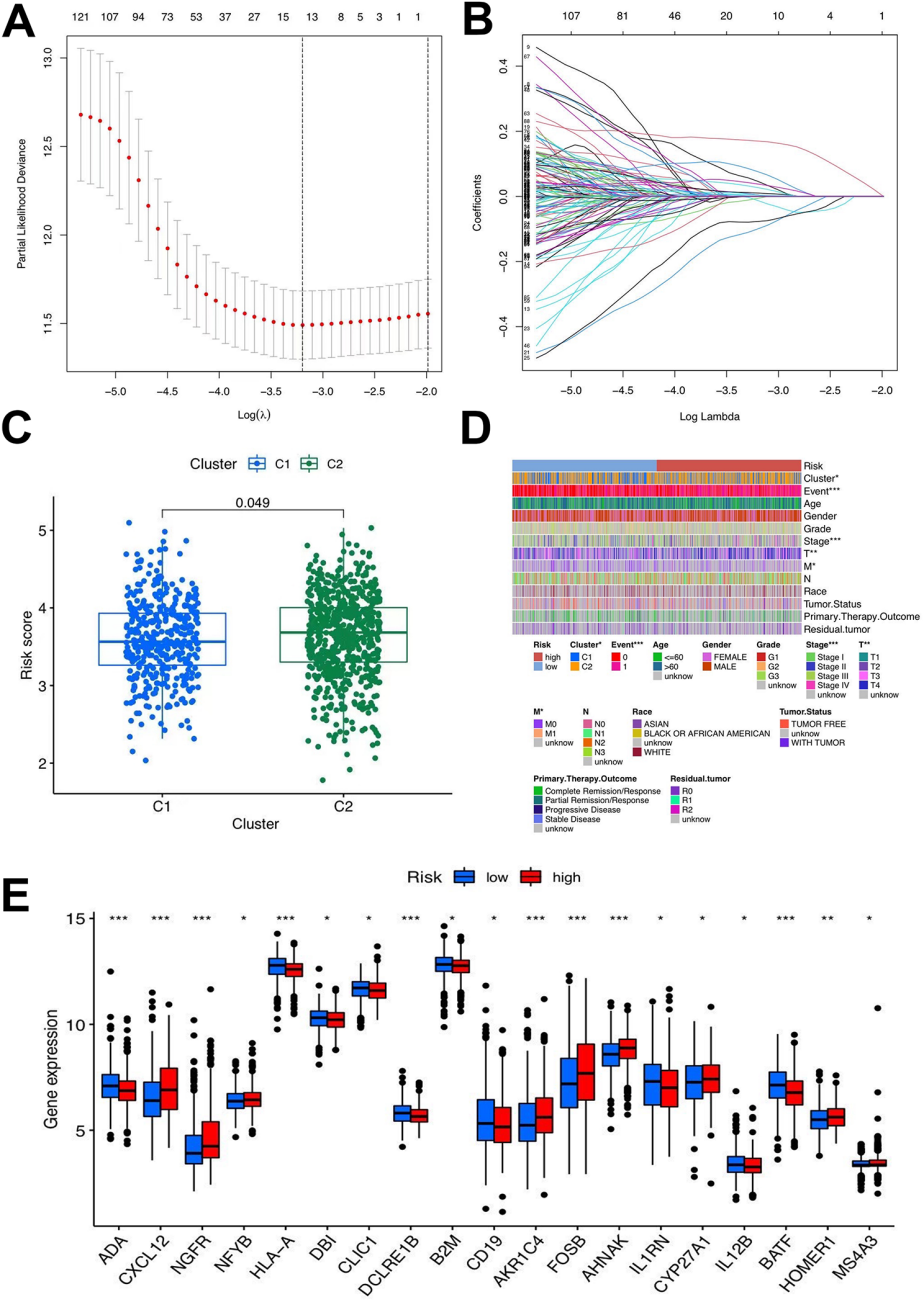
Selection of optimal prognostic signatures and constructure of risk signature in the training set. **a-b.** Least absolute shrinkage and selection operator(LASSO) regression analysis for prognostic genes. **c.** Differences in risk score among distinct gene clusters. **d.** Heat map regarding the correlation between the risk signature, molecular, genetic classification, prognosis and clinical features. **e.** Differences in the expression of Differential Expression (DEG) Analysis among the low-risk group and high-risk groups

LASSO and multivariate Cox regression analyses were employed, and 13 genes were selected from 178 genes by LASSO to construct signatures; six core genes (CD5, ABCA8, SERPINE2, ESM1, SERPINA5, and NMU) were screened from the DEGs to build the risk signature (Fig. 5a, b). Patients with STAD (*n* = 1043) were stochastically separated into a training group (*n* = 522) and a test group (*n* = 521) using the caret R package, in which the training group was employed to construct signatures. After the multivariate Cox regression analysis, the process of constructing the risk score was calculated as follows:

Risk score = (-0.3081*expression of CD5) + (0.18156*expression of ABCA8) +.

(0.2309*SERPINE2) + (0.2112*expression of ESM1) + (0.0970*SERPINA5) + (0.1116*NMU).

The outcomes of the risk-scoring system applied to all patients showed significant diversity. Patients with STAD and risk scores below the average standard in the gene cluster were assigned to the low-risk group (*n* = 549), whereas those exceeding the average standard were assigned to the high-risk group (*n* = 494). Overall, the risk score of patients with STAD in the T cell C2 group was higher than that in the other groups (Fig. 5c).

**Fig. 6:**
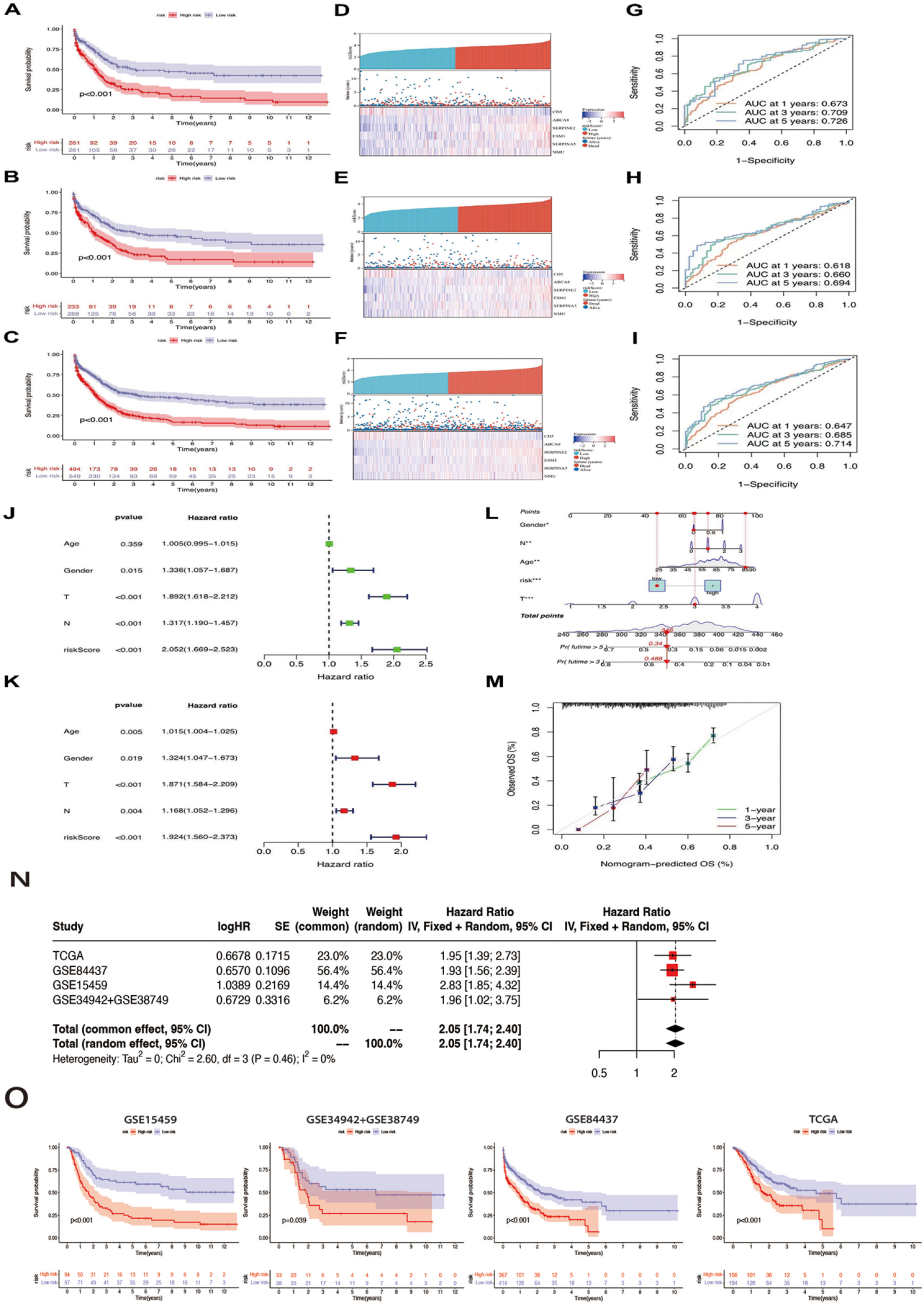
Validation of the risk signature in training, testing sets and the two combined. **a-c.** Kaplan-Meier survival analysis between the high- and low-risk score groups in training, testing sets and the two combined. **d-f.** Scatter plots showing the survival status of stomach adenocarcinoma (STAD) patients with increasing risk scores. Ranked dot plots indicating the risk score distribution. Heat maps showing the distribution of the six candidate genes. **g-i.** receiver operating characteristic (ROC) curves predicting the sensitivity and specificity of 1-, 3- and 5-year survival according to the risk signature. **j-k.** Univariate COX regression analysis in the merged cohort and multivariate COX regression analysis in the merged cohort. **l-m.** Nomogram and Calibration curves for prediction of 1-, 3-, and 5-year survival rate of STAD patients in the two sets. **n.**Prediction effects of univariate and multivariate cox regression analyses on individual cohorts. **o.** Survival analysis in individual cohorts. The cohort order is as follows: GSE15459, GSE34942 + GSE38749, GSE84437, TCGA

A heatmap was created, highlighting that tumor, node, and metastasis staging was elevated in the high-risk group (Fig. 5d). This conclusion also confirmed that C1 had a significantly better prognosis than C2. Gene expression in the two groups was visually analyzed to thoroughly investigate the association between the risk score and other parameters (Fig. 5e). When TCGA and GEO cohorts were merged, log2 was used for data with large values to eliminate batch effects (Additional figure S1f). Survival analysis was carried out in the training and test groups and the initial merged cohort to guarantee that the outcome of the obtained risk signature’s predictive ability could be confirmed. Similar survival advantage results were observed across all analyzed groups (Fig. 6a-c). In the low-risk group, the expression of T cell-related genes was relatively high, whereas in the other groups, the expression was low. The distribution of risk scores demonstrated that a lower risk score was associated with a higher survival probability and that the survival rate decreased with an increase in the risk score. From the distribution risk score curve, affordable T cell-related genes such as CD5 were higher in the low-risk group, whereas the inverse outcome was observed in the high-risk group. ABCA8, SERPINE2, ESM1, SERPINE2, and NMU expression levels were higher in the high-risk group, whereas an inverse outcome was noted in the low-risk group. The six core genes were distributed in the low- and high-risk groups within the training, testing, and total samples using heat maps. The probability of survival decreased as the risk score increased (Fig. 6d-f). To verify the veracity of the risk signature, we generated ROC curves (Fig. 6g-i). Univariate and multivariate Cox regression analyses of the combined cohort also verified that the risk characteristics could be independently applied as a prognostic signature for STAD (Fig. 6j, k). The 1-, 3-, and 5-year survival rates of patients could also be predicted using a contingency map containing risk scores and clinicopathological parameters (Fig. 6l). Based on the calibration chart, the previous line chart had characteristics similar to those of the calibration plot (Fig. 6m). We analyzed the predictive effects of univariate and multivariate Cox regression analyses on individual cohorts (GSE15459, GSE34942 + GSE38749, GSE84437, and TCGA) (Fig. 6n). A survival analysis was performed to test the predictive effect of the signature on individual cohorts. In all cohorts, survival decreased with increasing risk scores (Fig. 6o).

### Characteristics of TME and results of immunotherapy

**Fig. 7:**
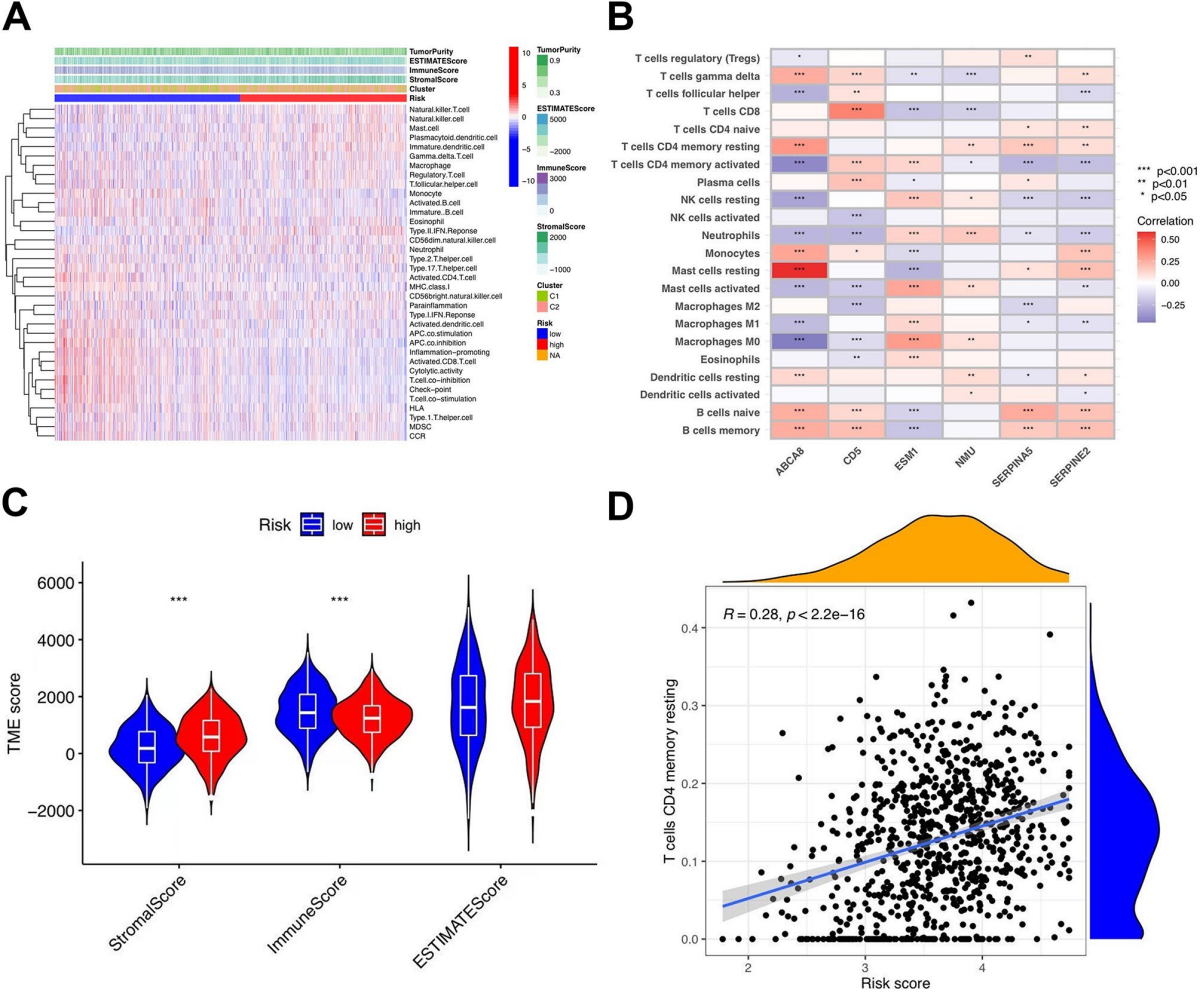
Immune annotation and correlation between T-cell and anti-PD-1/L1, anti-CTLA-4 immunotherapy. **(a)** Relations between tumor purity, ESTIMATE score, immune score, stromal score, and different stomach adenocarcinoma (STAD) phenotypes. **(b)** Correlation between the abundance of immune cells and six candidate genes. **(c)** Violin plot illustrating the result of ESTIMATE analysis. **d.**Relationships between the risk signature and cancer stem cell (CSC) index

**Fig. 8:**
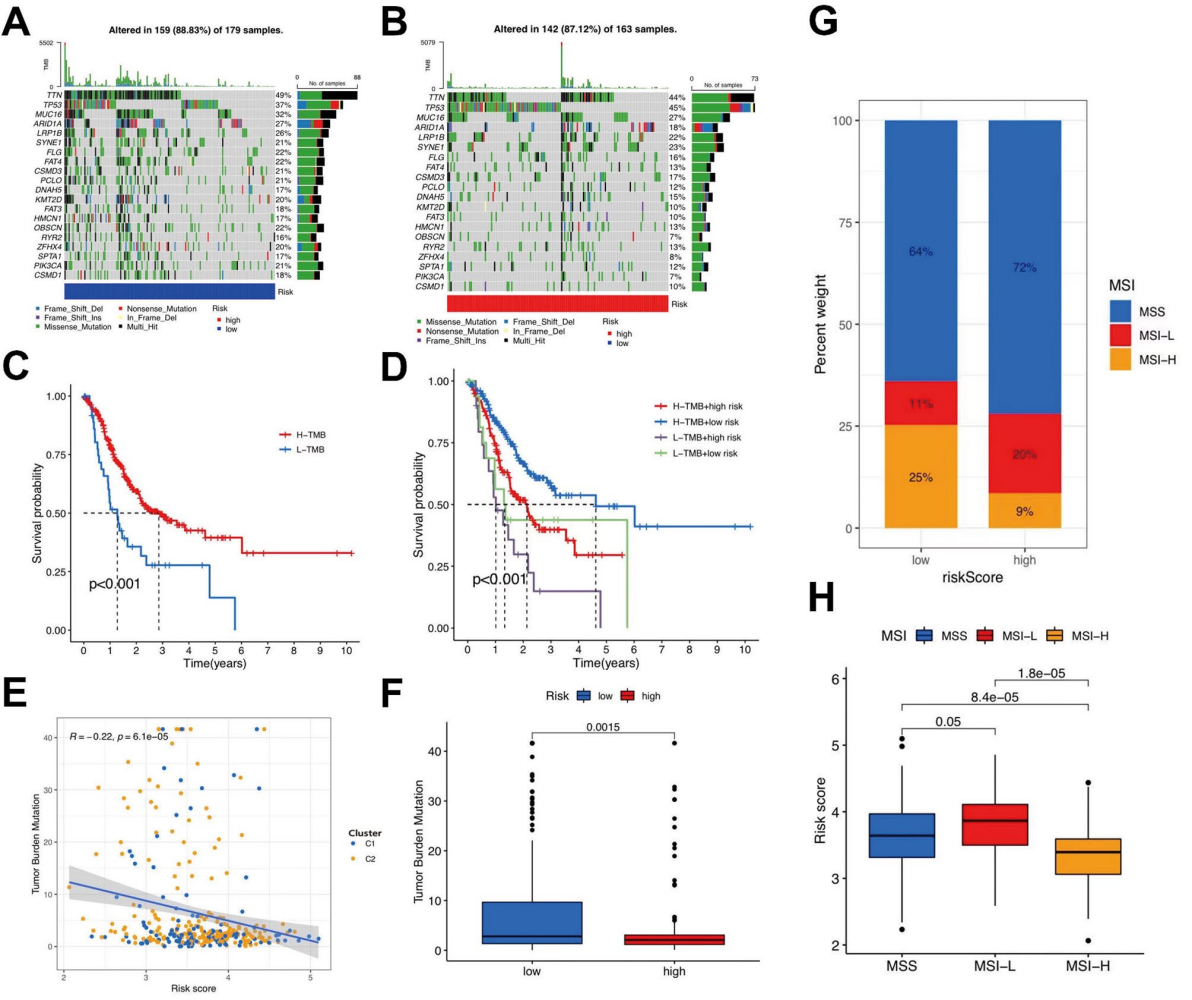
Exploration of the association between the tumor somatic mutation, Microsatellite Instability(MSI) and risk signature. **a-b.** The waterfall plots of tumor somatic mutation constructed by those with low- and high-risk scores, respectively. **c.** Survival analysis on stomach adenocarcinoma (STAD) samples with high and low tumor mutational burden(TMB). **d.** Survival analysis on STAD samples with different TMB and risk score. **e.** Relationships between TMB and risk score based on T-cell clusters, respectively. **f.** Distribution of STAD samples with low- and high-risk score in TMB. **g-h.** Relationships between risk signature and MSI

The correlation between the clusters and risk was visualized using a scatter diagram and heatmap (Fig. 7a). The results showed mast cells, monocytes, and type II interferon responses were positively linked to the risk score. T-cell inhibition, activated CD8 + T cells, activated B cells, and other immune cells were significantly negatively correlated. The matrix and estimate scores in the low-risk group were lower; however, there was no significant difference between the two groups (Fig. 7b). The interstitial and estimate scores showed marked variation between the low- and high-risk subgroups (Fig. 7c). The survival ratio and degree of purity of tumor cells in the high-risk group were significantly higher than those in the low-risk group. In STAD, the comprehensive risk score and cancer stem cell (CSC) index values were used to comprehensively assess the link between risk markers and CSC (Fig. 7d). The study displayed that risk score was positively associated with the CSC index (*R* = 0.28; *p* < 2.2e-16), demonstrating that the higher the risk score, the more obvious the differentiation grade of the stem cells. Subsequent analysis to explore the immune infiltration condition of T cell-related core genes was performed by studying the distribution disparity of somatic cell mutations between the low- and high-risk score groups using the maftools package (Fig. 8a, b). We performed a survival analysis of TMB and risk scores in different groups to forecast the prognosis of patients with STAD, and the outcome illustrated that the low-risk score and high-TMB groups had the highest survival rates (Fig. 8c, d). We also assessed the degree of association between TMB and the risk score, as well as between T-cell clusters and gene clusters (Fig. 8e). The results further demonstrated that low-risk scores correlated with high TMB. In addition, the TMB of clusters C2 and B were higher than those of the other two clusters. The low-risk group, compared to the high-risk group with a higher mutation load, was more extensive, which was consistent with the above findings and confirmed by subsequent TMB quantitative analysis (Fig. 8f). We also analyzed microsatellite instability (MSI) in the low- and high-risk subgroups to further assess the capacity of risk markers to predict ICB responses in patients (Fig. 8g). The rate of MSI was higher in the low-risk group than in the high-risk group, suggesting that immunotherapy and clinical therapy were more effective in this subgroup. The association between the risk characteristics and MSI was further confirmed (Fig. 8h).

### Immune escape and immunotherapy analysis

**Fig. 9:**
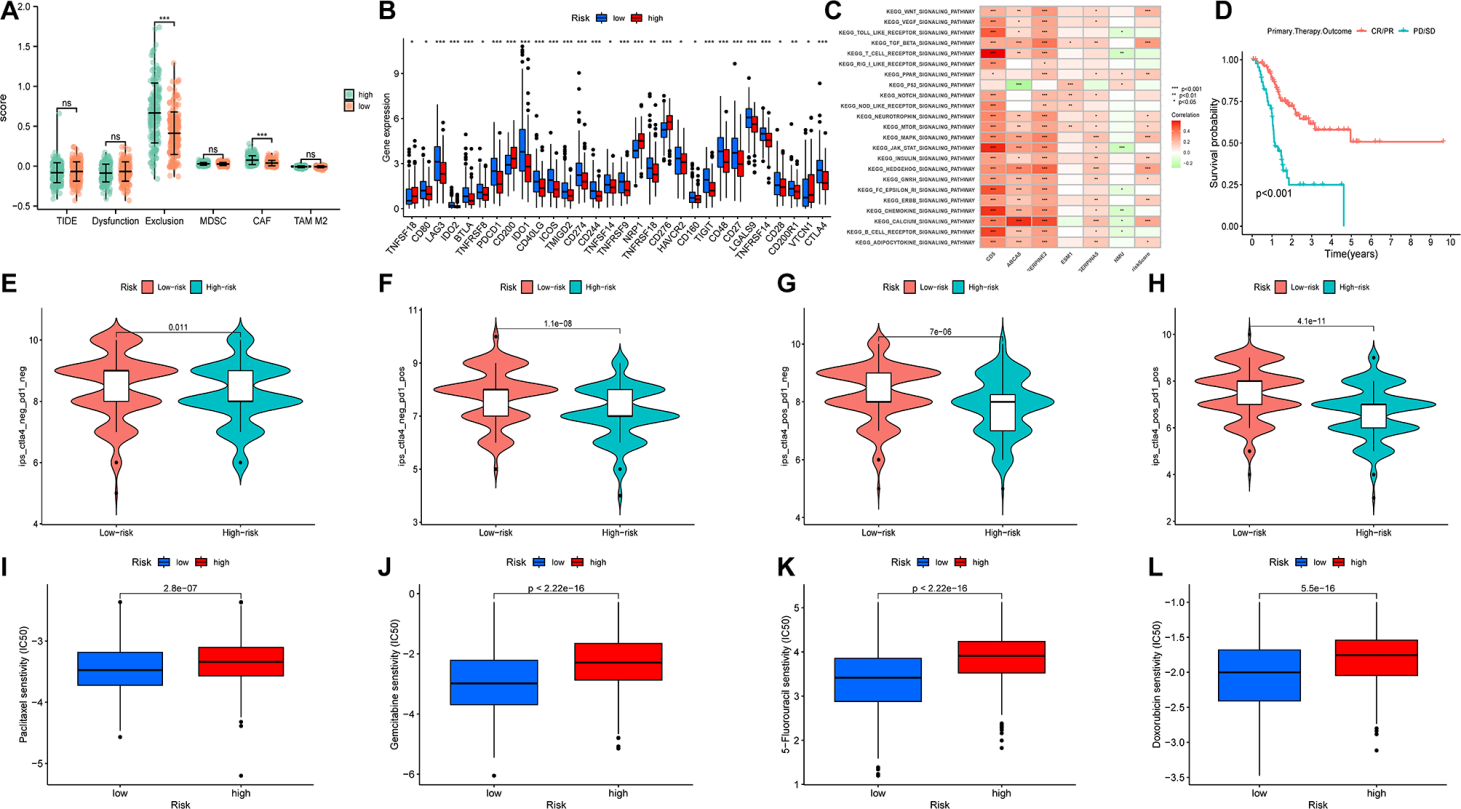
Immunotherapy and Drug sensitivity analysis. **(a)** Box plot suggesting the difference of response towards immunotherapy between high- and low-risk score group. **(b)** Differences in the expression of T-cells among the low-risk group and high-risk groups. **(c)** gene set variation analysis(GSVA) analysis of 6 core genes of the signature. **(d)** survival after immunotherapy. **e-h.** The efficacy of immunotherapy for high- or low- risk groups. **i-l.** Drug sensitivity of Paclitaxel, Gemcitabine, 5-Fluorouracil and Doxorubicin

The high-risk group exhibited a high immune escape rate and poor immunotherapeutic efficacy (Fig. 9a). Through the analysis of immune cell differentiation, it can be seen that the immune expression of the PDCD1 and CD274 high-risk group was low. Based on the data obtained, PD1 and PD-L1 were not very effective in the immune treatment (Fig. 9b). According to the characteristic value of the sample in each module and the characteristics of the sample, correlation analysis was performed to identify two modules related to specific traits: KEGG T-cell receptor signaling pathway and KEGG B-cell receptor signaling pathway were positively correlated in CD8 T cells (Fig. 9c). The treatment effect was observed using survival analysis in each group. The CR/PR group exhibited the highest probability of survival (Fig. 9d). Estimate analysis revealed a significant gap between the matrix and estimate score between the low- and high-risk subgroups (Fig. 9e-h). The results indicated that the survival rate of tumor cells in the high-risk group was significantly higher than that in the low-risk group, accompanied by poorer immune efficacy. The half-maximal inhibitory concentrations (IC50) of paclitaxel, gemcitabine, 5-fluorouracil, and doxorubicin were all higher in the high-risk groups, indicating potential drug resistance (Fig. 9i-l).

### Reference to an external cohort for validation

**Fig. 10:**
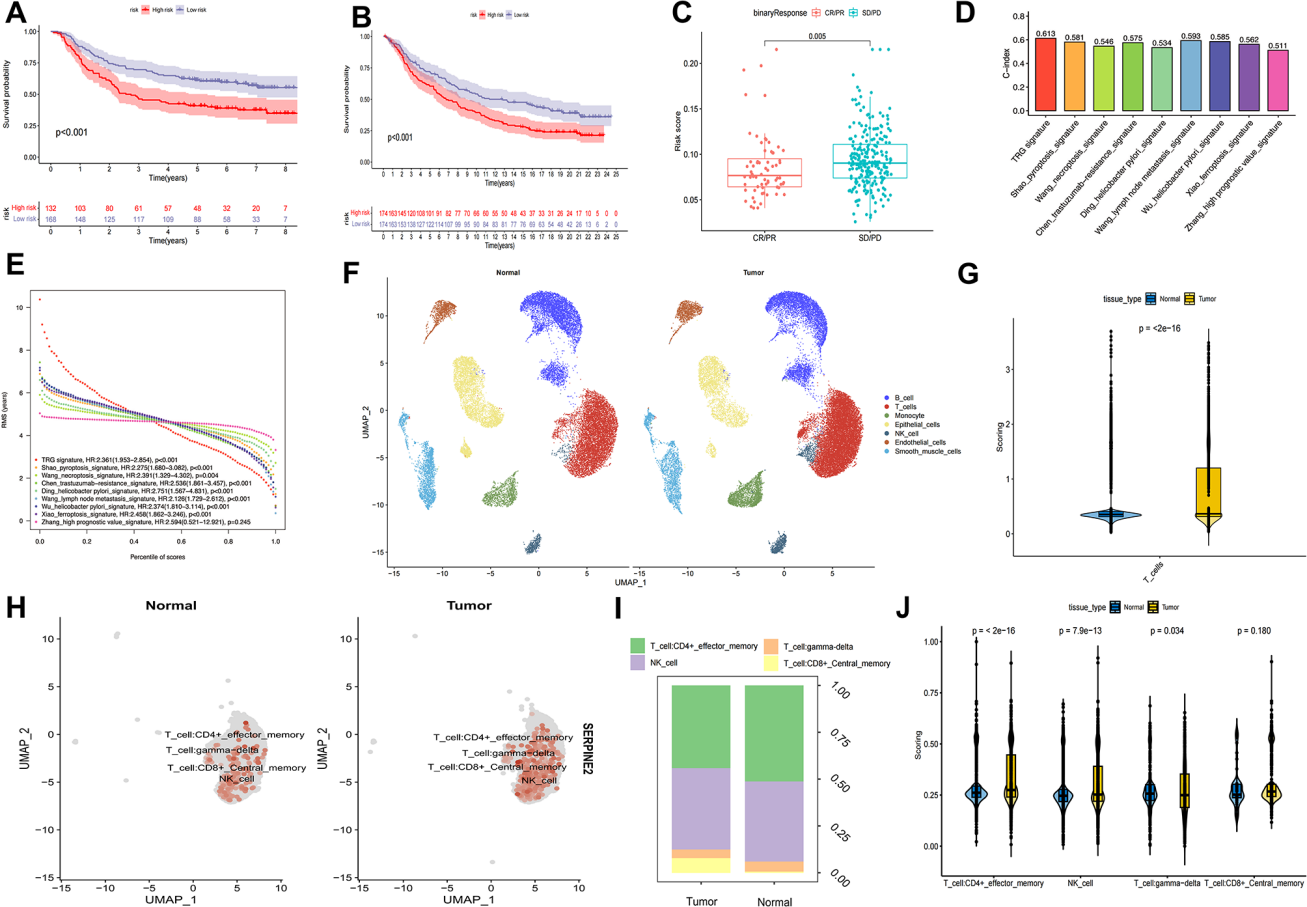
Cohorts of IMV210 and GSE62254 and external signatures for the validation of TRG-signature, and further scRNA analysis based on GSE18394. **a, b.** Kaplan-Meier survival analysis between the high- and low- risk score groups. In GSE62254 and IMV210,respectively. **c.** Binary response in risk score among CR/PR and SD/PD. **d.** C-index of different signatures. **e.** Restricted Mean Survival (RMS) Curves for different Signature Values. **f.** The distribution of different cells in gastric cancer and adjacent areas. **g.** The total gene enrichment score in T cell. **h.** Expression of SEPRINE2 gene in T cells. **i.** Ratio map of T cell subsets. **j.** Gene enrichment score in subset

Survival analyses were performed on the external IMV210 and GSE62254 cohorts to confirm the predictive power of this risk marker in validating the survival advantage of the low-risk group. Similar results were observed in all pooled analyses (Fig. 10a, b). The binary response system showed that the SD/PD risk scores were significantly higher than those of CR/PR (Fig. 10c).

### Signature discrimination

We compared our T cell-related gene signature with other published signatures [7, 26–33], which yielded a C-index of 0.613, which was higher than other signatures published within the last 3 years (Fig. 10d). According to restricted mean survival, it also achieved a higher accuracy of survival estimation in the validation datasets (HR: 2.361; *p* < 0.001; Fig. 10e). ROC curves and survival analyses were generated for comparison (Additional figure S2).

### Comprehensive analysis of scRNA

After quality control, 54,274 cells were labeled to show their distribution (Fig. 10f). Enrichment analysis suggested that the T-cell content in tumors was significantly higher compared to normal tissues (Fig. 10g). Furthermore, the T-cell subpopulations were divided into gamma delta, CD4+, NK, and CD8 + T cells. SERPINE2 was highly expressed in tumors, mainly in CD4 + T cells and NK cells (Fig. 10h, i). The signature score was higher for CD4 + T cells and NK cells of tumors and lower for gamma delta T cells (Fig. 10j).

### Immunohistochemistry and Youjiang cohort analysis

**Fig. 11:**
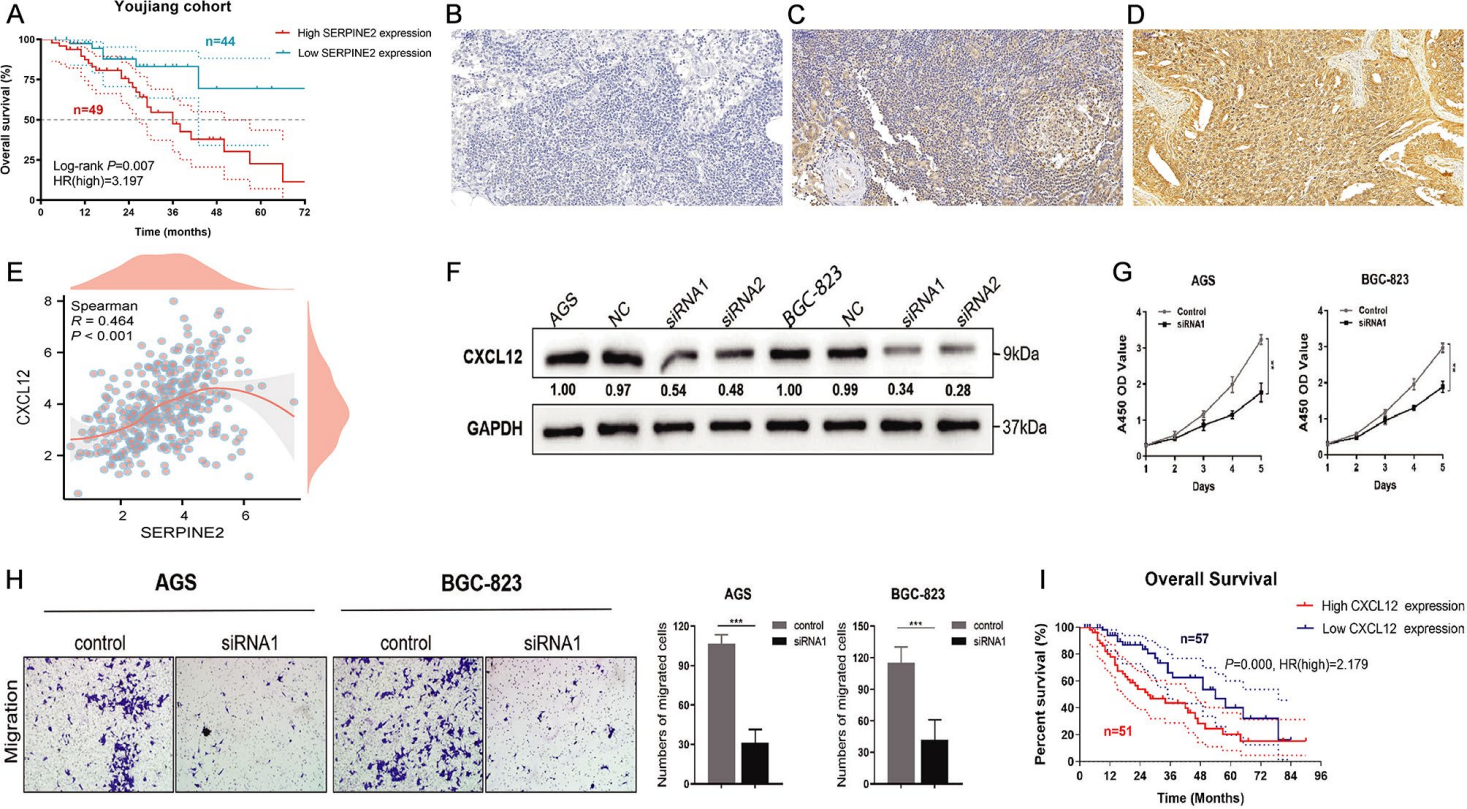
Clinical cohort and experimental exploration of SERPINE2 and CXCL12 in GC. **a** Youjiang cohort of overall survival. **b-d.** Low, medium and high expression in immunohistochemistry of samples from Youjiang cohort. **e.** Relationship between SERPIN2 and CXCL12. **f.** Down-regulated of CXCL12 in AGS and BGC-823 cells. **g.** CCK-8 assay, down-regulated level of CXCL12 expression significantly reduce the proliferative ability of GC cells. **h.** Transwell cell migration assay analyse the down-regulation level of CXCL12 expression of GC cell. **i.** Survival analyses conducted on patients with different CXCL12 expression level. *P < 0.05, **P < 0.01, ***P < 0.001

Based on immunohistochemistry, the Youjiang cohort was divided into Low SERPINE2 (*n* = 44) and High SERPINE2 (*n* = 49) groups, and samples with lower SERPINE2 expression exhibited better OS (HR = 3.197; *p* = 0.007; Fig. 11a-d). As shown in Fig. 11e, the SERPIN2 and CXCL12 levels were significantly correlated.

### Down-regulation of CXCL12 inhibited the proliferation and migration of AGS and BCG-823 cells

To investigate the effects of CXCL12 in vitro, we first validated the downregulation of CXCL12 expression in AGS and BCG-823 cells (Fig. 11f). According to the results of the CCK-8 assay, the knockdown of CXCL12 significantly reduced the proliferative viability of AGS and BCG-823 cells compared to the control group (Fig. 11g). Furthermore, the transwell migration assay indicated that the knockdown of CXCL12 expression markedly suppressed the metastatic ability of AGS and BCG-823 cells (Fig. 11h). Overall, the downregulation of CXCL12 significantly inhibited the proliferation and migration of AGS and BCG-823 cells.

### Association between survival and GC subtypes defined by CXCL12 expression

To determine the effect of CXCL12 on patient survival, we analyzed the survival of patients with different CXCL12 expression levels. Survival analysis showed that patients with higher CXCL12 expression had worse OS than those with lower CXCL12 expression (Fig. 11i). These data suggest that the analysis of CXCL12 expression yields different subtypes of GCs. Specifically, our results suggest that lower CXCL12 expression levels are associated with improved survival in patients with GC.

## Discussion

The incidence of STAD is decreasing in most developed countries [34]; however, the number of deaths due to the disease is increasing [35]. Currently, treatment for gastric cancer is not satisfactory [36]. Moreover, more than half of the patients diagnosed with gastric cancer cannot be treated surgically at the time of diagnosis [37]. The lack of treatment for gastric cancer also leads to rapid disease progression and increased mortality. Previous studies have investigated the correlation between genes and carcinogenesis in various cancers, including GC [38, 39]. Multiple types of genomic damage, including the activation of oncogenes and inactivation of tumor suppressor genes, are factors that cause gastric cancer [40]. Its anti-tumor effects are characterized by the highly coordinated actions of many genes. Owing to the inadequacy of current medical technology, only one or two genotypes have been evaluated [41].

The present study aimed to build signatures to study the effect of certain genes on gastric cancer using LASSO and multivariate Cox regression analyses. In this study, LASSO and multivariate Cox regression analyses were applied to build signatures to study the effect of certain genes on gastric cancer. Six core genes (CD5, ABCA8, SERPINE2, ESM1, SERPINA5, and NMU) were selected from the DEGs to establish risk marker signatures. The results showed that The risk score of T cell Cluster C2 was significantly higher than that of T cell Cluster C1, and the prognosis of C2 was significantly better than that of CI. We also performed validation using an external cohort, which further confirmed that our phenotypic classification of T cell-associated gene mutations was meaningful. In addition, we preliminarily found that the signature genes were closely correlated with STAD, which provides valuable clues for further research on immunotherapy targets for STAD.

Initially, we selected four genes (IL12B, B2M, HLA-A, and CD19) for further study, among which CD19 and IL12B showed a significant survival advantage in the predictive analysis. Autologous CD19-targeted CAR T-cells could significantly help treat blood cancer [42]. Epidemiological studies have shown that IL-12B is associated with an increased incidence of cervical cancer [43]. The TME cannot be ignored during tumor development [44] as it contains many different cell types, such as endothelial and fibroblast [45]. Tumor-infiltrating immune cells can directly or indirectly participate in immune responses, thereby affecting the prognosis of patients with tumors [46]. For example, dendritic cells can capture antigens emitted by tumors, while Effector T cells (CD8+) and TAMs can lyse and phagocytose tumor cells.

Additionally, helper T cells (CD4+) limit the immune response [47]. Inhibition of these cytokines can strengthen the anti-tumor effect of tumor-infiltrating lymphocytes and further improve their clinical therapeutic effect [48, 49]. A recent study also confirmed that T helper cells are effective prognostic immune cells, which is correlated with further studies on gastric cancer [50]. Based on immunological and drug sensitivity analyses, we found that the high-risk group had a higher probability of immune escape and was generally resistant to first-line chemotherapy, indicating insensitivity to these treatment methods.

The scRNA results suggested that, compared to normal tissue, T-cell infiltration in GC was more abundant, mainly composed of differentiated CD4 + T cells and NK cells, while gamma delta T cells with higher differentiation potential were fewer, indicating T-cell exhaustion in the tumors. SERPINE2, which had the highest score in the signature, was highly expressed in T cells from GC. We found a significant positive correlation between SERPINE2 and the T cell-related factor CXCL12 in our dataset. Previous studies indicated that CXCL12 interacts with T cells to reduce OS in patients with GC [51], while SERPINE2 promotes cell proliferation [52]. Correspondingly, we tested the effects of CXCL12 downregulation on cell proliferation and migration and found that CXCL12 significantly reduces promotional and migration potential in GC cell lines. Survival analyses performed for patients with different CXCL12 expression levels confirmed that patients with high CXCL12 expression levels had poor survival probability. However, our study not only confirmed the effects of CXCL12 on tumor cell promotion and metathesis, but also showed the potential value of CXCL12 in tumor treatment. Therefore, we preliminarily speculated that SERPINE2 affects CXCL12 through a potential pathway, thereby promoting T-cell exhaustion.

Numerous methods were employed to assist our signature in this study; however, there were still some shortcomings. Environmental, racial, economic, predictive, and follow-up factors influence OS [22]; this is a limitation of our study, and in a follow-up study, we will control for the variables for a further in-depth study.

## Conclusions

This work may contribute to the understanding of tumor immunity and provide new ideas for the personalized treatment of STAD.

### Electronic supplementary material

Below is the link to the electronic supplementary material.

Supplementary Material 1

## Data Availability

The datasets analysed during the current study are available in the TCGA, GSE15459, GSE34942, GSE38749, GSE84437, GSE62254 and IMV210 repository.

## Abbreviations

GC: Gastric cancer
STAD: Stomach adenocarcinoma
TME: Tumor microenvironment
scRNA: Single-cell ribonucleic acid
DEG: Differentially expressed genes
DO: Disease ontology
GO: Gene ontology
KEGG: Kyoto Encyclopedia of Genes and Genomes
CNV: Copy number variations
PPI: Protein-protein interaction
ICIC: Inter-cell Interference Coordination
CSC: Cancer stem cell
MSI: Microsatellite instability
IC50: Half-maximal inhibitory concentrations
