# Supplementary material for: Comprehensive analysis of T-cell regulatory factors and tumor immune microenvironment in stomach adenocarcinoma
Source: BMC Cancer. 2024 May 7;24:570. doi: 10.1186/s12885-024-12302-w (PMC11077837; doi:10.1186/s12885-024-12302-w)
Supplement: Supplementary file 1 — Supplementary Material 1 [file 12885_2024_12302_MOESM1_ESM.doc]

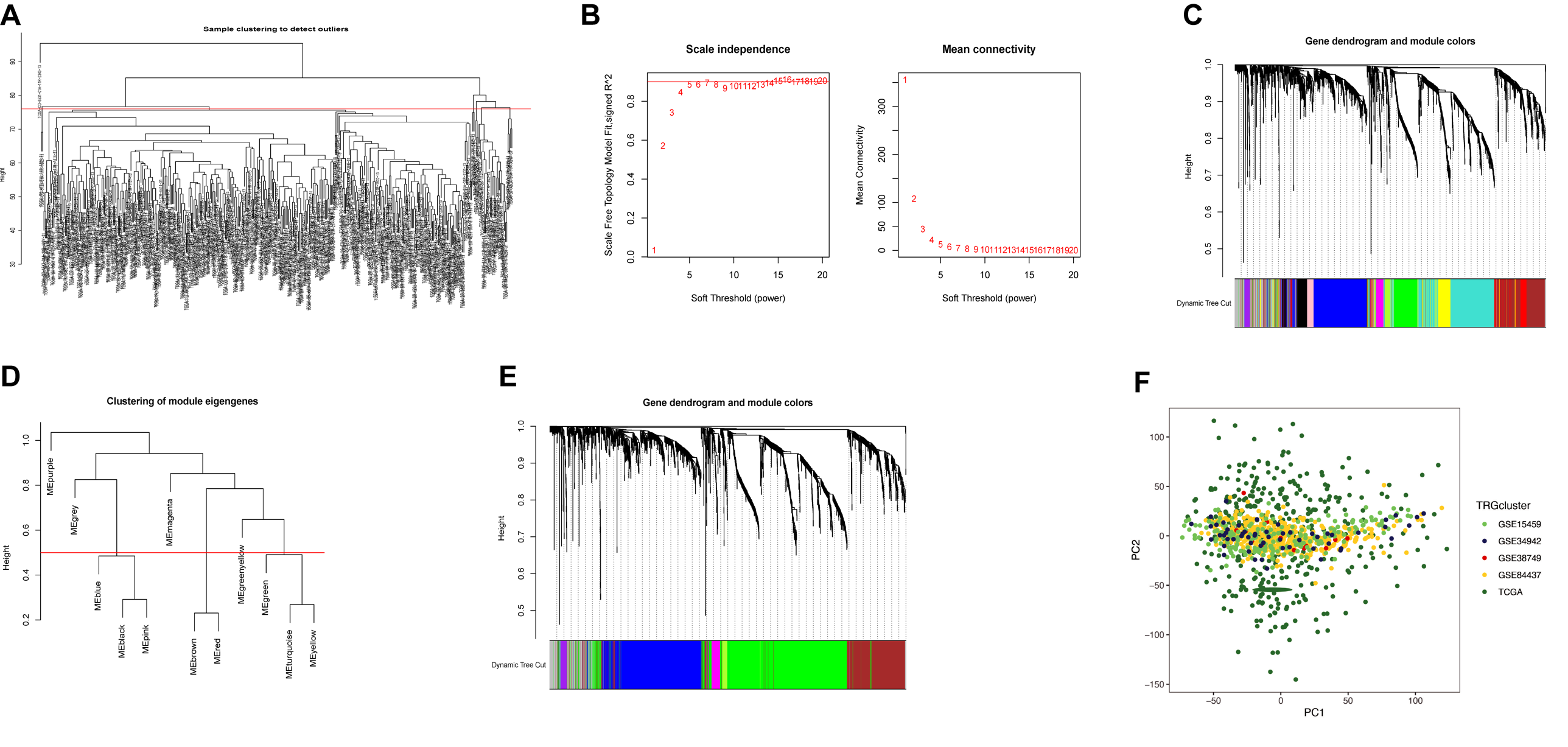


**FIGS1. Weighted correlation network analysis(WGCNA) co-expression analysis of stomach adenocarcinoma (STAD) samples.**

**a.** Sample clustering to detect outliers, red intercept line set according to sample dispersion. **b. Left picture:** Changes of correlation between m and n under different β values. **Right picture:** Under different β values, the mean of all gene connectivity. **c.** Gene dendrogram obtained by average linkage hierarchical clustering. **d.** Clustering of module eigengenes.  **e.** Gene dendrogram and module colors **f.** Eliminate batch effects before merging TCGA and GEO signatures.


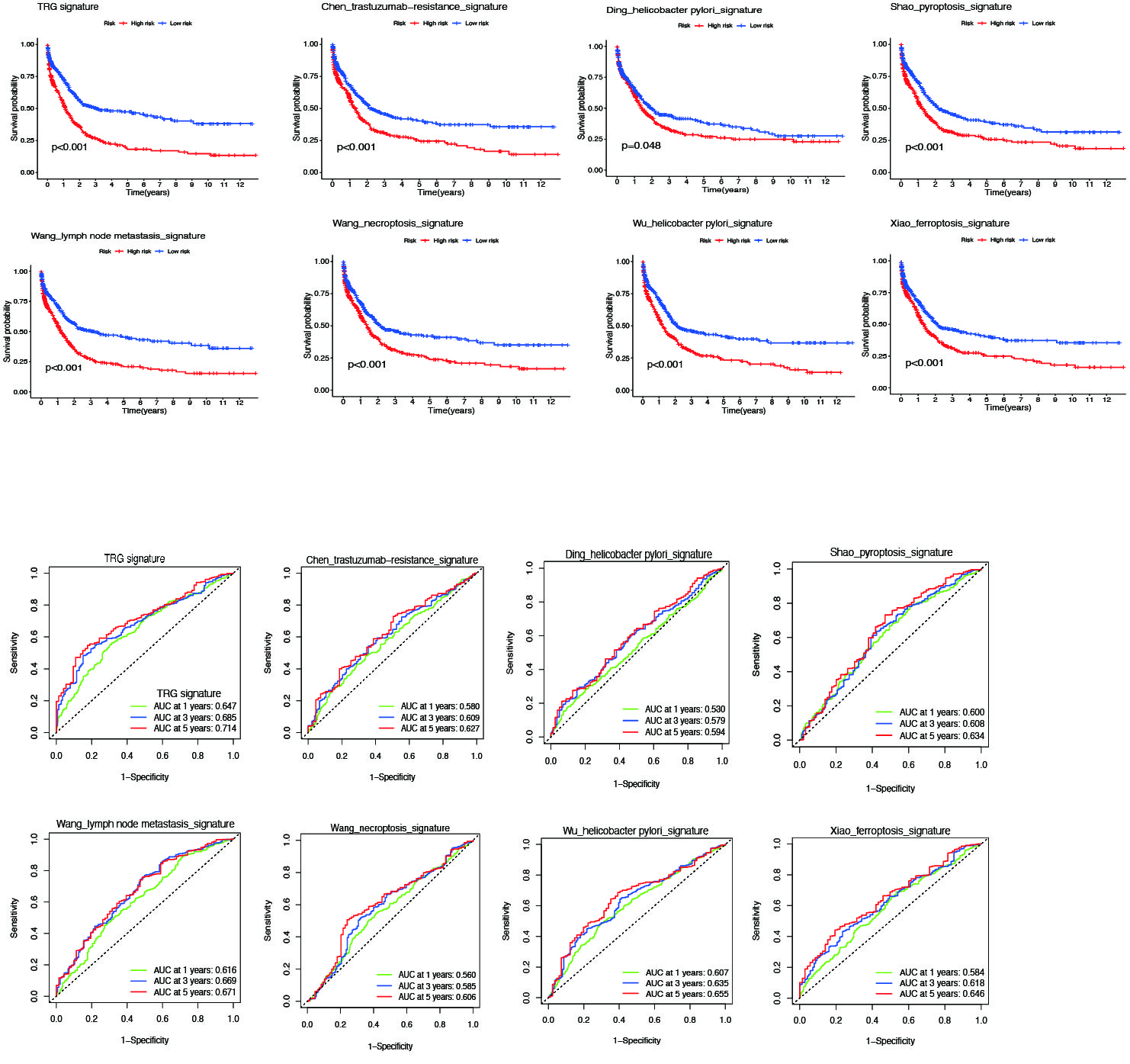


**FIGS2. ROC curves and survival analyses were generated for comparison with other published signatures.**

**
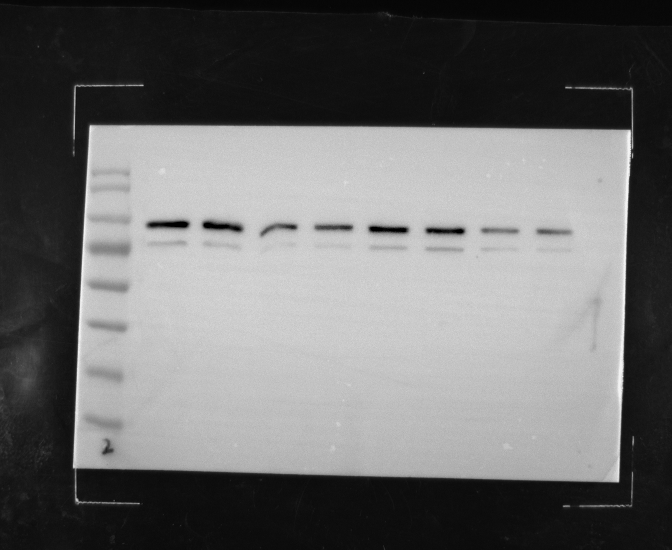
**

**Original blots of CXCL12**


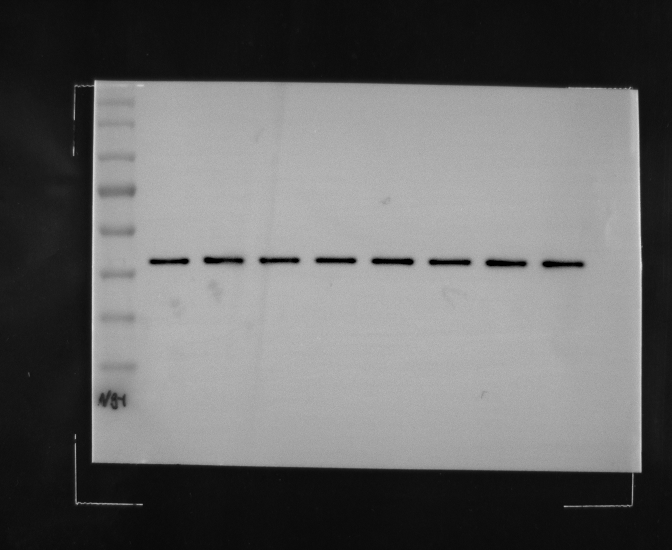


**Original blots of GAPDH**
